# Three easily-implementable changes reduce median door-to-needle time for intravenous thrombolysis by 23 minutes

**DOI:** 10.1186/s12883-019-1527-8

**Published:** 2019-11-26

**Authors:** Demi Tran, Zhu Zhu, Mohammad Shafie, Hermelinda Abcede, Dana Stradling, Wengui Yu

**Affiliations:** 1 Department of Neurology, University of California, Irvine, CA USA; 2 Department of Neurology, Huashan Hospital, Fudan University, Shanghai, China; 3 Orange, USA

**Keywords:** Acute ischemic stroke, Intravenous thrombolysis, Door-to-needle time, Quality improvement

## Abstract

**Background:**

The benefit of intravenous thrombolysis (IVT) for acute ischemic stroke is time dependent. Despite great effort, the median door-to-needle time (DNT) was 60 min at the United States stroke centers. We investigated the effect of a simple quality improvement initiative on DNT for IVT.

**Methods:**

This is a single-center study of patients treated with IVT between 2013 and 2017. A simple quality improvement initiative was implemented in January 2015 to allow the Stroke team to manage hypertension in the emergency room, to make decision for IVT before getting blood test results unless patients were taking oral anticoagulants, and to give IVT in the CT suite. Baseline characteristics, DNT and outcomes at hospital discharge were compared between pre- and post-intervention groups.

**Results:**

Ninety and 136 patients were treated with IVT in pre- and post-intervention groups, respectively. The rate of IVT was significantly higher in the post-intervention group (20% vs. 14.4%, *p =* 0.007). The median DNT with interquartile range (IQR) was reduced significantly by 23 min (63[53–81] vs. 40[29–53], *p* < 0.001) with more patients in the post-intervention group receiving IVT within 60 min (81.6% vs. 46.7%) and 45 min (64.0% vs.17.8%). There was no significant difference in symptomatic intracerebral hemorrhage rate (1.5% vs. 1.1%), modified Rankin Scale 0–1 (29.4% vs. 23.3%), and hospital mortality (7.4% vs. 6.7%) between the 2 groups.

**Conclusions:**

Three easily-implementable quality improvement initiative increases IVT rate and reduces DNT significantly without increasing the rate of IVT-related complications in our comprehensive stroke center.

## Introduction

Intravenous thrombolysis (IVT) with tissue-type plasminogen activator (tPA) is the proven medical therapy for acute ischemic stroke (AIS), with faster administration resulting in better outcomes [1, 2]. However, the diagnosis and treatment of AIS are often delayed for various reasons, including lack of pre-notification, unclear last-known-well (LKW) time, waiting for blood test results, lag in getting CT scan and reports of imaging findings, holdup in mixing tPA or transportation between emergency room (ER) and CT scan suite, management of uncontrolled hypertension, performing CT angiography and CT perfusion, and determination of eligibility [3–10].

Researchers at Helsinki University Central Hospital in Finland were able to implement measures to reduce delays and cut the median door-to-needle time (DNT) to 20 min with interquartile range (IQR) 14–32 min [4]. The Helsinki protocol was successfully replicated at the Royal Melbourne Hospital to reduce DNT to 25 (IQR, 19–48) minutes during business hours (8 AM to 5 PM Monday-Friday) in 2012 [5]. A few hospitals in Canada and Netherlands were also able to reduce median DNT to 25–37 min [6–9].

In contrast, the quality improvement endeavors have not worked out very well in the United States (U.S.). Despite the launch of American Heart Association (AHA)/American Stroke Association (ASA) Target: Stroke initiatives in 2010 and comprehensive stroke center (CSC) certification by the Joint Commission in 2012 [10, 11], the IVT rate and median DNT at the U.S. stroke centers remains suboptimal. A study of AIS patients registered in *Get With The Guidelines*-Stroke from October 2012 to April 2015 showed that the median DNT was 60 min with only 50% patients treated within 60 min [12]. The Target: Stroke phase II was launched in April 2014. The median DNT from 888 surveyed hospitals between June 2014 and April 2015 was still 56 (IQR, 42–75) minutes [13]. In a recent study comparing stroke care and outcomes between CSCs and primary stroke centers (PSCs) in the U.S. from 2013 to 2015, the median DNT was 52 (IQR, 39–70) minutes at CSCs and 60 (IQR, 47–83) minutes at PSCs [14]. The IVT rates were only 14.3 and 10.3%, respectively.

It appears that centralized hospital system in developed countries were able to implement quality improvement initiatives efficiently [4–9]. The healthcare system in the U.S. is decentralized with higher average annual IVT volume in CSCs than in PSCs (40 vs. 22) [14]. Due to overall low annual volumes and labor-intensive code stroke protocols, the stroke centers in the U.S., including CSCs and PSCs, appear to have significant logistic restraints in implementing comprehensive quality improvement initiatives 24/7 [11–13].

Due to limited numbers of vascular neurologists and insufficient staff support, our stroke center was unable to implement some of the Stroke: Target phase 1 strategies during 2010 and 2014. We started to focus on the 3 easily-implementable changes in January 2015 to improve stroke care at our CSC. This study aimed to explore the effect and safety of this simple initiative.

## Methods

### Patients and design

This is a single-center study approved by the University of California Irvine Institutional Review Board. Acute Ischemic stroke is characterized by the sudden loss of blood circulation to an area of the brain, resulting in a corresponding loss of neurologic function. Consecutive patients with LKW within 4.5 h were evaluated for thrombolysis. Patients receiving IVT at the University of California Irvine CSC between January 2013 and December 2017 were included. A simple quality improvement protocol was developed in January 2015: 1) To allow the stroke team to manage hypertension in the ER promptly to keep BP goal < 185/110 mmHg. Patient was initially treated with labetalol or hydralazine 10 mg IV pro re nata and then IV nicardipine infusion at 2.5–15 mg/hour to keep steady BP control. Vital signs were measured every 15 min. 2) To make decision for IVT before getting blood test results (complete blood count, comprehensive metabolic panel, cardiac enzymes and coagulation) unless patients were taking anticoagulants. All patients were carefully interviewed and examined to rule out history of coagulopathies, thrombocytopenia, and severe metabolic abnormalities before intravenous thrombolysis and t-PA would be immediately stopped when any potential contraindication for IVT was found during thrombolysis. All patient got finger stick for glucose measurement in the ambulance or Emergency Department (ED). Severe hypoglycemia was excluded before thrombolysis through finger stick glucose measurement. The benefit and risk of IVT were discussed with patients and/or their relatives, and written informed consent was not required American Heart Association guidelines [1]. 3) To give IVT in the CT suite.

The patients were divided into pre-intervention (January 2013 to December 2014) and post-intervention (January 2016 to December 2017) groups, with one-year washout period (January 2015 to December 2015) allowing for the full implementation of the initiative.

The following information was collected and compared between the pre- and post- intervention groups: age, gender, past medical history (hypertension, diabetes, hyperlipidemia), National Institutes of Health Stroke Scale (NIHSS) score at admission, DNT, symptomatic intracranial hemorrhage (sICH), in-hospital mortality, and modified Rankin Scale (mRS) at hospital discharge. sICH was defined as parenchymal hemorrhage type 2 (dense blood clot exceeding 30% of the infarct volume with substantial space-occupying effect) on CT within 36 h [2].

### Statistical analysis

Continuous variables were described by mean ± standard deviation or median with interquartile range (IQR) based on the results of normality testing. Categorical variables were expressed by counts with percentages. Baseline characteristics and outcomes at discharge were compared between pre- and post- intervention groups by t test or Wilcoxon rank-sum test for continuous variables and χ2 test for categorical variables. The proportions of sICH, functional independence (mRS 0–1), poor outcome (mRS 5–6), and in-hospital mortality were further compared between the 2 groups using multivariate logistic regression analysis after adjusting for age, hypertension, diabetes, hyperlipidemia and baseline NIHSS score. Analyses were performed using SPSS software (IBM, Version 23). A 2-tailed value of *P* < 0.05 was considered statistically significant.

## Results

A total of 1305 patients with AIS were admitted to our medical center during the study period and 294 of them received and completed IVT. After excluding 68 patients treated during the transitional year of the quality improvement initiative, there were 90 patients in pre-intervention group (from January 2013 to December 2014) and 136 in post-intervention group (from January 2016 to December 2017). The demographics and treatment benchmarks of the 2 groups are shown in Table 1. Compared with pre-intervention group, significantly more patients were treated with IVT in the post-intervention group (20.0% vs. 14.4%; OR = 1.39; *p* = 0.007). There was no difference in patient age, gender, history of hypertension or diabetes between the 2 groups. The post-interventional group had significantly higher rate of hyperlipidemia and lower NIHSS scores at admission than pre-intervention group. The patients with minor stroke (NIHSS ≤4) appeared to be more likely to receive IVT in the post-intervention group (27.2% vs. 10.0%, *p* < 0.001).

**Table 1:** Demographics and clinical features of patients in the pre-intervention and post-intervention groups

Variables	Pre-intervention	Post-intervention	OR (95% CI)	*p* value
Age	70 + 16	71 + 17	–	0.745
Male	44 (48.9)	68 (50.0)	1.04 (0.61–1.78)	0.870
Hypertension	62 (68.9)	107 (78.7)	1.67 (0.91–3.06)	0.097
Diabetes	22 (24.4)	46 (33.8)	1.58 (0.87–2.87)	0.132
Hyperlipidemia	29 (32.2)	62 (45.6)	1.76 (1.01–3.07)	0.045
SBP ≥ 185/110 mmHg	21(23.3)	41 (30.1)	1.44 (0.81–2.54)	0.215
NIHSS	14 (8–21)	7 (4–16)	–	< 0.001
NIHSS ≤4	9 (10.0)	37 (27.2)	3.36 (1.53–7.38)	0.002
DNT	63 (53–81)	40 (29–53)	–	< 0.001
DNT < 60 min	42 (46.7%)	111 (81.6%)	5.08 (2.79–9.26)	< 0.001
DNT < 45 min	16 (17.8%)	87 (64.0%)	8.20 (4.31–15.3)	< 0.001

Data are n (%), mean ± standard deviation, or median (interquartile range)

*OR* Odds ratio, *CI* Confidence interval, *SBP* Systolic blood pressure, *NIHSS* National Institues of Health Stroke Scale, *DNT* Door-to-needle time

**Fig. 1:**
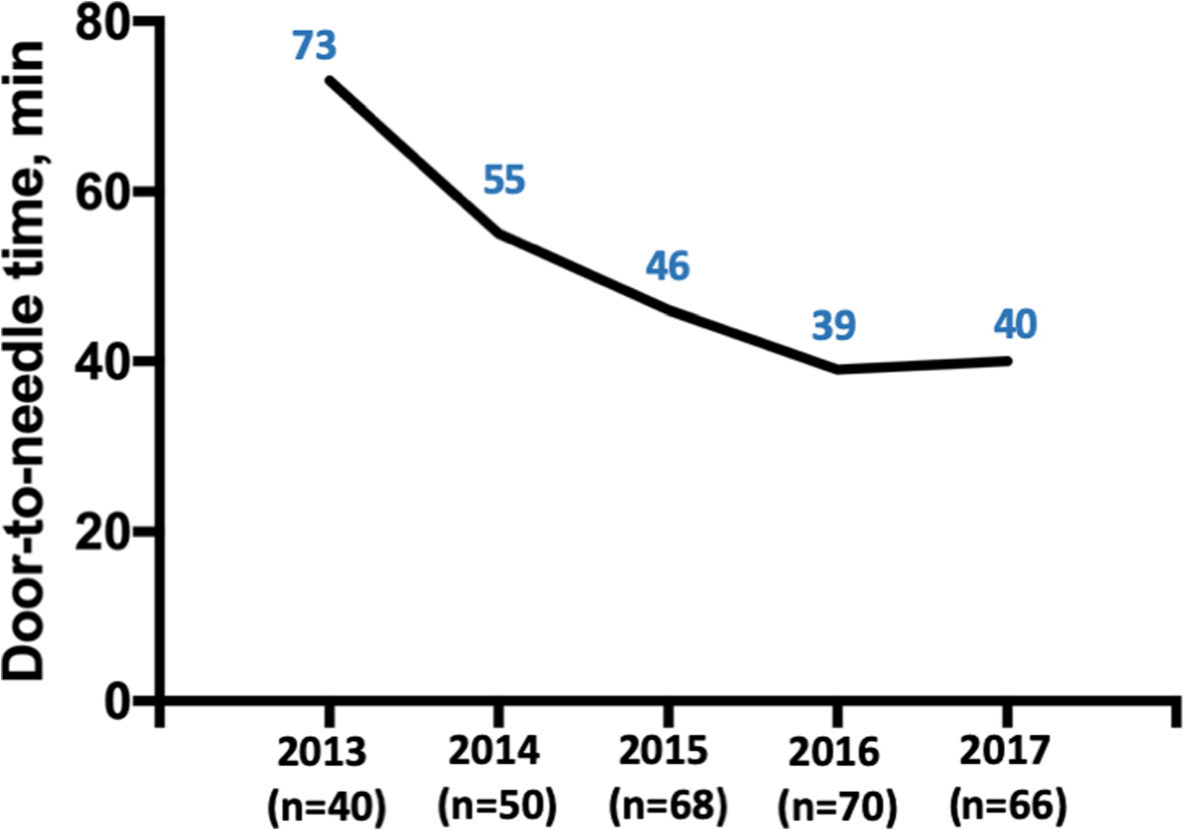
Median door-to-needle time from 2013 to 2017

The median DNT was reduced by 23 min from 63 (IQR, 53–81) minutes in the pre-intervention group to 40 (IQR, 29–53) minutes in the post-intervention group (*p* < 0.001), with a trend of continuous improvement from 2013 to 2017 (Fig. 1). In addition, significantly more patients in the post-intervention group received IVT within 60 min (81.6% vs. 46.7%, *p* < 0.001) and 45 min (64.0% vs. 17.8%, *p* < 0.001) than in the pre-intervention group. Of note, there was no significant difference in DNT between patients with minor (NIHSS ≤4) and major (NIHSS > 4) stroke (median 50 min, IQR 25–75 min vs. median 50 min, IQR 36–71 min; *p* = 0.317).

**Fig. 2:**
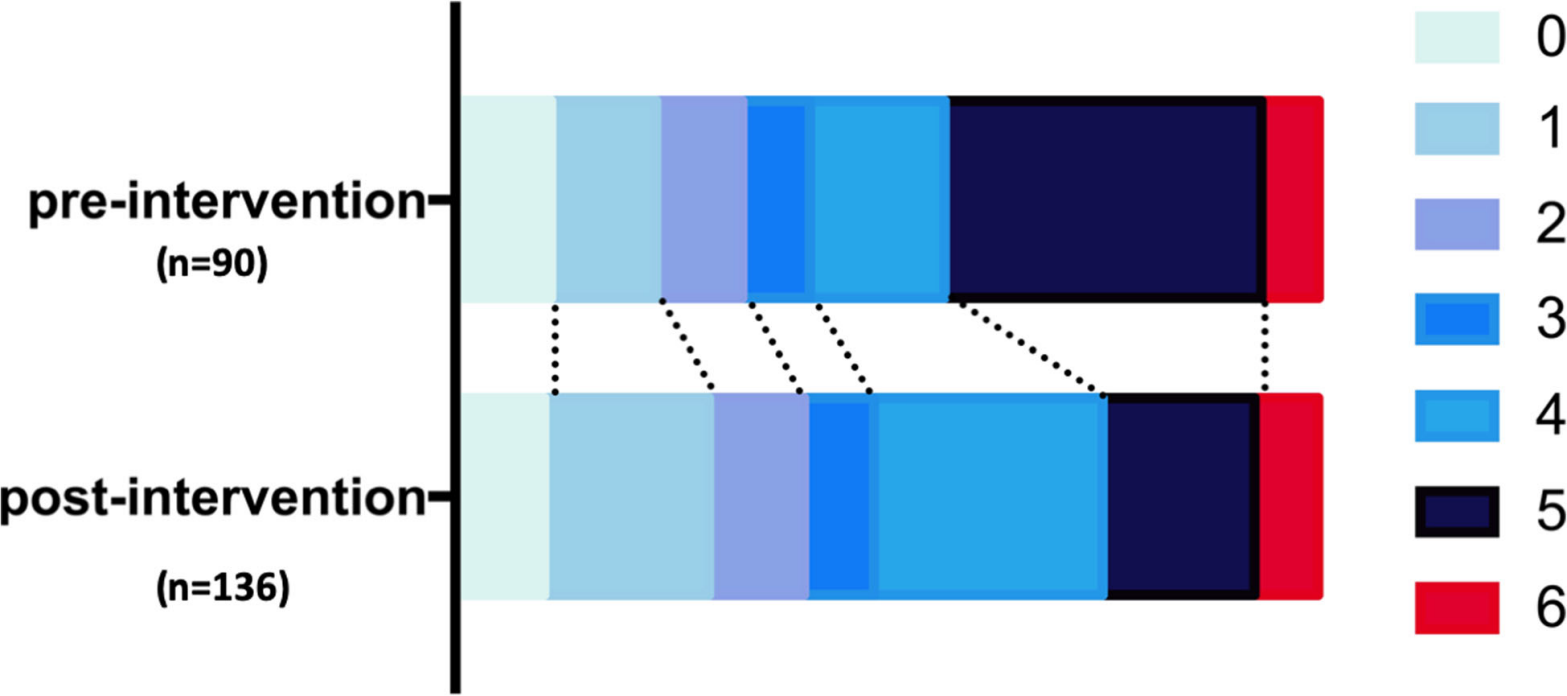
mRS at discharge in pre- and post- intervention groups. (χ2 test and multivariate logistic regression analysis adjusting for age, hypertension, diabetes, hyperlipidemia and baseline NIHSS score; unadjusted *p* = 0.054, adjusted p* = 0.067)

The rates of sICH and functional outcomes at hospital discharge are summarized in Table 2. There was no significant difference in the rates of sICH (1.5% vs. 1.1%), functional independence (mRS 0–1, 29.4% vs. 23.3%), and hospital mortality (7.4% vs. 6.7%) between the 2 groups. There was a trend of better functional outcome (more patients with mRS 0–1 and significantly less patients with mRS 5–6) in the post-intervention group (Table 2 and Fig. 2). However, in the multi-variate regression models, there was insignificant difference between the 2 groups after adjusting for age, hypertension, hyperlipidemia, diabetes and NIHSS score at admission.

**Table 2:** Outcomes in the pre- and post-intervention groups at discharge

Outcomes	Patients receiving IVT		OR (95% CI)	*p* value	Adjusted OR (95% CI)^a^	*p* value^a^
	Pre-intervention (*n* = 90)	Post-intervention (*n* = 136)				
sICH	1 (1.1)	2 (1.5)	1.33 (0.12–14.87)	0.817	1.15 (0.11–12.39)	0.911
mRS 0–1	21 (23.3)	40 (29.4)	1.37 (0.74–2.53)	0.314	1.01 (0.50–2.04)	0.983
mRS 5–6	39 (43.3)	34 (25.0)	0.44 (0.25–0.77)	0.004	0.53 (0.27–1.05)	0.068
mortality	6 (6.7)	10 (7.4)	1.11 (0.39–3.17)	0.844	1.33 (0.44–4.03)	0.619

Data are n (%)

*sICH* Symptomatic intracranial hemorrhage, *OR* Odds ratio, *CI* Confidence interval, *IVT* Intravenous thrombolysis, *mRS* Modified Rankin Scale

^a^Adjusted for age, hypertension, diabetes, hyperlipidemia and baseline NIHSS

## Discussion

With a simple and easily-implementable quality improvement initiative, we have increase IVT rate from 14.4 to 20.0% and reduced median DNT by 23 min to 40 min. Our results are better than recently reported benchmarks of 14.3% IVT rate and 52 min DNT at the 134 CSCs in the U.S. [14].

Despite significant improvement, our results are still suboptimal compared with the benchmarks from centralized Hospital Systems in other developed countries [4–9]. For example, the hospital district of Helsinki and Uusimaa has a population of 1.6 million and a centralized regional emergency medical service (EMS) [4, 15]. All patients deemed as candidates for stroke therapies are transported with high priority and pre-notification to the Helsinki University Hospital, which is the only 24/7 neurology service to provide care for AIS. As a high volume and centralized Stroke center, the Helsinki University Hospital was very efficient due to thorough training for all EMS and ED staff, and long-standing experience [4, 15].

In contrast, our CSC, the only academic medical center in Orange County, California, is one of the 9 stroke receiving centers serving a population of 3.19 million [16]. In such decentralized healthcare system, it is very challenging for all of the 9 stroke receiving centers to implement comprehensive protocols to achieve the fastest DNT for IVT [4–11, 14].

Our quality improvement initiative is easily-implementable, effective and safe. Uncontrolled hypertension is one of the most frequently reported factors causing delayed DNT [5, 8, 17]. In a single center study, uncontrolled hypertension was associated with more than 30 min delay in DNT [16]. Per AHA/ASA guidelines, IVT should be held until BP is less than 185/110 mmHg [1]. In the pre-intervention group, severe hypertension was managed by the ER physicians. During post-intervention period, stroke team was managing hypertension in the CT suite and ED without any delay as soon as patient was deemed to be eligible for IVT. This simple change effectively minimized hypertension-related delay for IVT.

Waiting for blood testing results is another common reason for delay up to 60 min in some eligible patients [5, 18]. Previous studies reported extremely low rates of unidentified coagulopathies and thrombocytopenia that would have been a contraindication for IVT [18–20]. Therefore, we implemented the initiative for IVT administration without waiting for blood test results unless patients were taking anticoagulants or had history of severe thrombocytopenia. There was no significant difference in the rate of sICH between pre-intervention and post-intervention groups. No patient suffered sICH from IVT due to undiagnosed coagulopathy. In addition, the rates of sICH in our cohort were much lower than reported in clinical trials [2], confirming the safety of our simple initiative.

Another change we made in practice in January 2015 was to give IVT in the CT suite. As CT imaging is an indispensable diagnostic tool for decision-making for IVT, shortening the CT imaging-to-needle time may significantly improve DNT [4, 5, 21, 22]. Our data confirmed that giving IVT in the CT suite minimizes the delay from CT to needle time without significant risk of complications.

Of note, the initial NIHSS scores in the post-intervention group was significantly lower than that in the pre-intervention group. There were numerous possibilities to explain why more patients with non-disabling stroke (NIHSS ≤4) during the post-intervention group. Previous studies showed longer DNT in patients with minor stroke, possibly due to higher chance of atypical symptoms, delayed neurology notification and diagnosis [23, 24]. The early diagnosis and decision making from better implementation of the Stroke: Target strategies in the post-intervention group would naturally increase IVT for patients with minor strokes. In addition, better stroke aware, risk management and stroke prevention in recent years may have also decreased the ratio of patients with more severe stroke.

The strength of our study is the use of 3 easily-implementable changes to reduce DNT without additional infrastructure cost or undue burden on the stroke team and ED staff. This simple initiative is effective, safe and easily replicable at other CSCs and PSCs in the U.S. and other countries.

This study has some limitations. First, it is a single center study. We used pre-intervention period as historic control. Our post-intervention data could be affected by unmeasurable confounding factors and gradual improvement in stroke care due to better training and experience. Second, although we had a one-year transition period for full implementation of the 3 changes, there might be incomplete adherence during the post-intervention period. Since incomplete adherence to the changes would likely lead to prolonged DNT, it is possible that the simple changes could work better if the initiative was applied all the time effectively. Third, as the aim of this study is to investigate the effect of a simple quality improvement initiative on DNT for IVT, some factors associated with outcomes at discharged were not included (eg, early ischemic changes on imaging, large artery occlusions, thrombectomy treatment). Finally, we have not addressed other hurdles in delaying DNT, such as point of care coagulation testing for patients taking anticoagulants, moving directly from the EMS stretcher to CT scanner [4, 7, 15]. Additional easily-implementable changes at decentralized healthcare system may significantly reduce DNT at CSCs and PSCs in the U.S.

## Conclusions

We demonstrated that three easily-implementable changes increase IVT rate and reduce DNT for IVT significantly without increasing the rate of IVT-related complications in our CSC.

## Data Availability

The data will be available from the corresponding author on reasonable request.

## Abbreviations

AHA: American Heart Association
AIS: Acute ischemic stroke
ASA: American Stroke emergency medical service
ER: Emergency room
IQR: Interquartile range
IVT: Intravenous thrombolysis
LKW: Last-known-well
mRS: Modified Rankin Scale
NIHSS: National Institutes of Health Stroke Scal
PSC: Primary stroke center
SD: Standard deviation
sICH: Symptomatic intracranial hemorrhage
tPA: Tissue-type plasminogen activator
